# Metastatic melanoma in an esophagus demonstrating Barrett esophagus with high grade dysplasia

**DOI:** 10.1186/1756-0500-6-457

**Published:** 2013-11-13

**Authors:** Dimitri G Trembath, Nicholas J Shaheen, Stacey O’Neill, Karen Weck, Kevin G Greene

**Affiliations:** 1 Department of Pathology and Laboratory Medicine, University of North Carolina at Chapel Hill, Chapel Hill, NC 27514, USA; 2 Department of Medicine and Epidemiology, Division of Gastroenterology and Hepatology, University of North Carolina at Chapel Hill, Chapel Hill, NC 27514, USA

**Keywords:** Barrett, Esophagus, Metastases, Melanoma, BRAF

## Abstract

**Background:**

Metastatic melanoma involving the esophagus is rare; the occurrence of metastatic melanoma in a background of Barrett esophagus is rarer still. We report a case of an 80 year-old male who presented to our institution for workup of Barrett esophagus with high-grade dysplasia and who proved to have metastatic melanoma occurring in the background of Barrett esophagus, the first report of this kind, to our knowledge, in the English literature.

**Case presentation:**

An 80 year-old Caucasian male was diagnosed at an outside institution with Barrett’s esophagus with high grade dysplasia and presented to our institution for therapy. The patient underwent endoscopic mucosal resection using a band ligation technique of an area of nodularity within the Barrett esophagus. Microscopic examination demonstrated extensive Barrett esophagus with high-grade dysplasia as well as a second tumor which was morphologically different from the surrounding high-grade dysplasia and which was positive for S-100, HMB 45 and Melan-A on immunohistochemistry, consistent with melanoma. Further workup of the patient demonstrated multiple radiologic lesions consistent with metastases. Molecular studies demonstrated that the melanoma was positive for the 1799T>A (V600E) mutation in the *BRAF* gene. The overall features of the tumor were most consistent with metastatic melanoma occurring in a background of Barrett esophagus with high-grade dysplasia.

**Conclusion:**

This case demonstrates a unique intersection between a premalignant condition (Barrett esophagus with high grade dysplasia) and a separate malignancy (melanoma). This report also shows the utility of molecular testing to support the hypothesis of primary versus metastatic disease in melanoma.

## Background

Melanoma is the most common tumor that metastasizes to the gastrointestinal tract, most frequently to the small bowel, while primary melanoma occurs most frequently in the anorectum, followed by the oral cavity, esophagus, stomach, small bowel, gallbladder, and large bowel [1,2]. Despite this, both primary and metastatic melanoma of the esophagus are rare entities with primary esophageal melanoma accounting for less than 1% of all primary esophageal malignancies and esophageal metastatic melanoma accounting for less than 5% of all melanomas metastatic to the gastrointestinal tract [2].

Barrett esophagus (BE) is a premalignant condition characterized by the replacement of the usual squamous epithelium of the distal esophagus by columnar-type mucosa which can be seen at endoscopy and which demonstrates intestinal metaplasia when biopsied [3]. The prevalence of BE in the general population varies from 1.6 to 5.6% depending upon the group studied [4]. Gastroesophageal reflux disease (GERD) is a strong risk factor for BE, and studies of subjects with chronic GERD symptoms demonstrate a prevalence of BE ranging from 5 to 15% [4]. The amount of acid exposure correlates with the risk and segment length of BE; other risk factors for BE include age, sex and ethnicity [4].

Patients with BE are at an increased risk for esophageal adenocarcinoma with an average incidence rate at 0.5% per year [4]. Increased segment length of BE (> 3 cm) is associated with an increased risk of carcinoma compared to short segment BE (< 3 cm) [3]. The development of dysplasia in BE frequently precedes the development of adenocarcinoma, with patients demonstrating a stepwise progression from low grade dysplasia (LGD) to high grade dysplasia (HGD), to intramucosal adenocarcinoma and finally to adenocarcinoma. Patients who develop HGD are at significantly increased risk for the development of adenocarcinoma, with studies placing the incidence of adenocarcinoma at between 5.6 to 6.6% per year [5]. Treatment for patients with BE consists of surveillance endoscopy and treatment of GERD, if present. Management of patients with HGD is more controversial with three possible strategies: surgical esophagogastrectomy, frequent surveillance endoscopy or endoscopic therapy [4].

Despite the frequent endoscopic surveillance of patients with BE, reports of BE with a concurrent second esophageal malignancies are rare; we report a case of an 80 year-old male who presented to our institution for management of BE with HGD. Subsequent evaluation demonstrated BE with HGD and a large nodule of melanoma invading into the submucosa. Our discussion reviews the differences between primary and metastatic melanoma of the esophagus and the rarity of collision tumors in the esophagus. To our knowledge, this is the first case in the English literature describing metastatic melanoma occurring in conjunction with BE and is the first to include molecular studies of the melanoma.

## Case presentation

### Case

**Figure 1:**
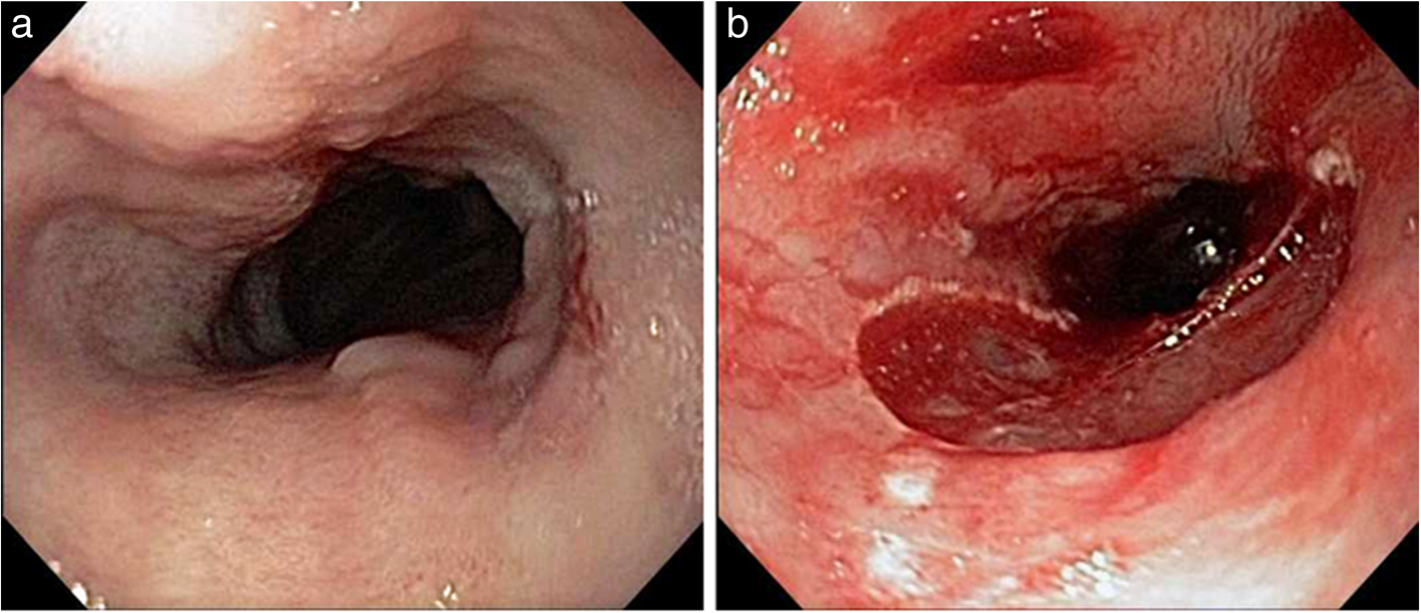
Endoscopic images demonstrating the area of BE with nodularity before (a) and after (b) resection.

An 80 year-old Caucasian male was referred to our institution in November 2011 for consideration of endoscopic therapy of BE with high grade dysplasia. Previously, the patient had underwent an endoscopy at an outside institution which demonstrated long segment BE from 20 to 30 cm, two discrete linear ulcers from 25 to 22 cm and a tiny hyper-pigmented erosion in the cardia. Esophageal biopsies at the outside institution demonstrated Barrett esophagus with high-grade dysplasia. At the time of endoscopy at our institution, there were esophageal mucosal changes secondary to established long segment BE extending from the upper extent of the gastric folds which were 35 cm from the incisors to the squamocolumnar junction, which was at 20 cm from the incisors. Nodularity was present from 24 to 28 cm. Mucosal resection was performed with the endoscopic resection within the BE limited to the area of nodularity. The resection was performed with a band ligation and snare resection technique (Duette Kit, Cook Medical, Bloomington, IN) with a total of four pieces resected (Figure 1a and b).

**Figure 2:**
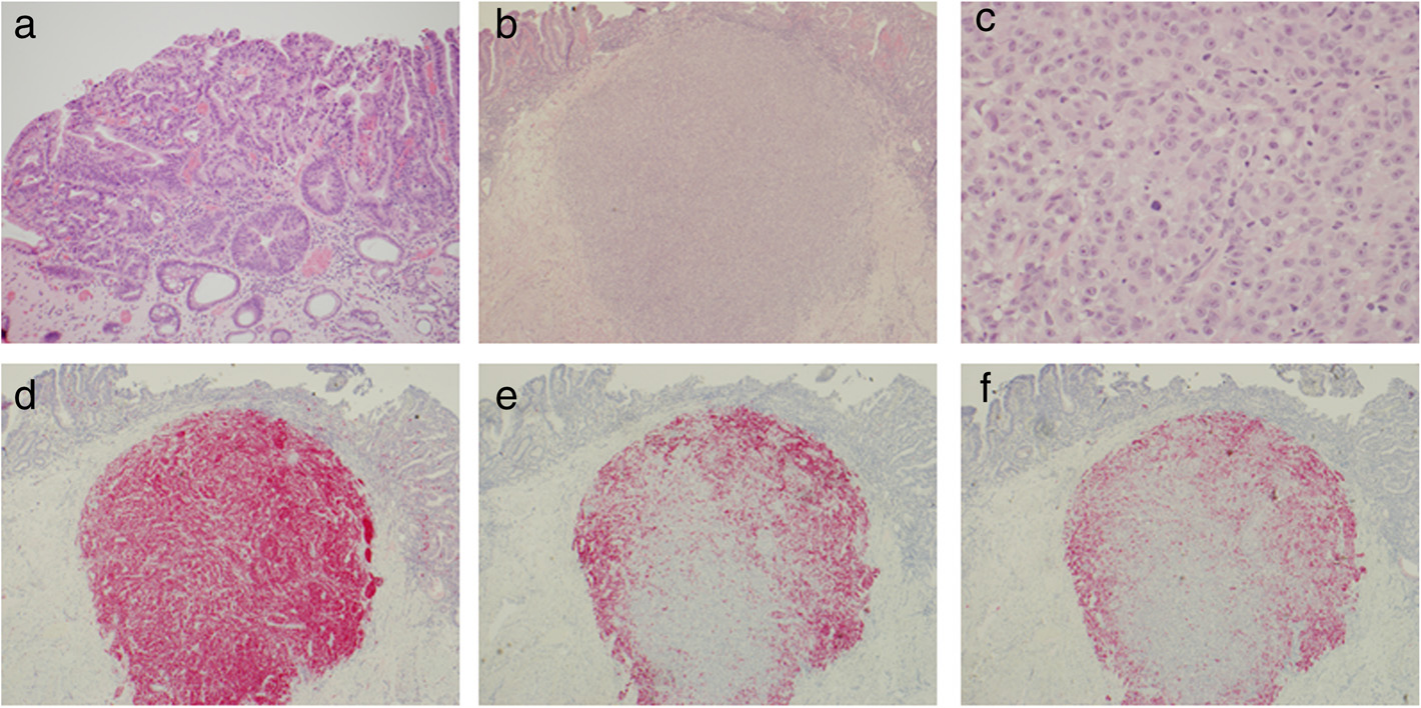
**Histological and immunohistochemical findings. a)** Barrett esophagus with high grade dysplasia present in the patient’s resection material (H&E, 100X). **b)** Second tumor nodule present within the mucosa and submucosa with distinct histologic features including **c)** large epithelioid cells with atypical nuclei with prominent nucleoli and frequent mitotic activity (H&E, 40X; H&E, 400X respectively). Immunohistochemical testing demonstrated the second tumor was positive for **d)** S-100, **e)** HMB-45 and **f)** Melan-A (each 40X).

Microscopic examination of the resected tissue demonstrated BE with HGD extending to the edge of the specimen. No invasive adenocarcinoma was identified (Figure 2a). Also present in one resected specimen was a well circumscribed nodule of tumor cells involving the submucosa with overlying mucosal ulceration (Figure 2b). A second, smaller nodule of tumor cells with prominent intra-cytoplasmic pigment was present in a separate section from the mucosal resection, involving the muscularis mucosa. These latter tumor cells were arranged in nests and demonstrated moderately atypical nuclei with frequent prominent nucleoli, surrounded by moderate amounts of eosinophilic cytoplasm (Figure 2c). Immunohistochemical stains were performed and demonstrated that the tumor nodule was negative for pan-cytokeratin and positive for S–100, HMB 45, and Melan-A (Figure 2d-f respectively). These findings were consistent with melanoma. A review of the H&E stained sections did not demonstrate the presence of any melanoma in situ in the overlying mucosa, which instead demonstrated high-grade dysplasia of BE. Overall, the findings were consistent with metastatic melanoma.

A subsequent PET scan demonstrated multiple areas of uptake in the bone marrow and skull consistent with widespread metastatic disease. Bilateral pleural effusions were present, as well as a small focus of uptake in the esophagus. Numerous lesions were identified in the liver and spleen, consistent with metastatic disease.

**Figure 3:**
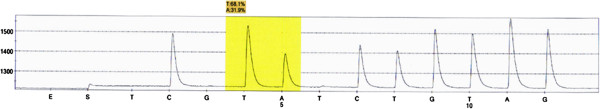
**Pyrosequencing results demonstrate the presence of a 1799T>A mutation in codon 600 of the *BRAF *gene.** The pyrogram shows a CAC>CTC change involving the second nucleotide position of codon 600, highlighted in yellow, in the reverse strand. Allele quantification reveals 68% mutant allele in the tumor sample. This nucleotide change results in a valine to glutamic acid substitution at amino acid position 600.

Molecular studies were performed on formalin fixed paraffin embedded tissue from the esophageal mucosal resection. Following tumor enrichment by macro-dissection of an area containing approximately 85% melanoma nuclei, molecular testing demonstrated the presence of a 1799T>A (V600E) mutation in *BRAF* (Figure 3). No mutations were found in exons 9, 11, 13, 17 and 18 of *KIT*. Due to the side effects of BRAF inhibitors, it was believed the patient would not benefit from this form of therapy. After discussion about other potential palliative therapy, the patient's family decided on hospice care.

## Discussion

Melanoma of the esophagus is a rare entity, both in primary and metastatic forms. Garfinkel provided one of the first reports of primary melanoma of the esophagus in 1952 and de la Pave *et al.* demonstrated the presence of melanocytes in esophageal epithelium in a small number of autopsy patients with esophagitis in 1963, providing a possible explanation for the development of primary melanomas in this location [6,7]. Diagnostic criteria for primary melanoma of the esophagus includes the presence of in-situ melanoma, a radial growth phase, melanocytosis, and mixed epithelioid and spindle cell morphology, in the context of no history of cutaneous melanoma [2,8]. Additionally, some authors have suggested that metastases to other anatomic sites or organs are important for distinguishing between primary esophageal melanoma and metastatic melanoma of the esophagus [2].

Collision tumors between BE and other malignancies are relatively rare. There have been reports of adenocarcinoma in BE arising with synchronous squamous cell carcinoma, small cell carcinoma, and other primary neuroendocrine tumors [9-11]. While there are existing case reports of primary melanoma of the esophagus, our review of the literature could only demonstrate two cases describing melanoma of the esophagus occurring in a background of BE. Both cases describe the melanoma as a primary lesion [8,12]. In reviewing our case, the histology is most consistent with metastatic melanoma rather than a primary lesion. There is no evidence of melanoma in-situ in the overlying mucosa, the tumor does not demonstrate a mixed epithelioid and spindle cell morphology, and the patient demonstrates the presence of additional metastatic lesions involving the bone marrow, liver and spleen. While no primary cutaneous melanoma could be demonstrated on physical exam, it may be that the primary melanoma has regressed, particularly given the patient's age. Appropriate immunohistochemical stains supported a diagnosis of melanoma and demonstrated that the tumor was distinctly different from the surrounding BE, which itself demonstrated features of high-grade dysplasia.

As with many tumors, the advent of modern molecular technology has greatly expanded our understanding of the genomic and protein basis behind many tumors. The discovery of constitutively activating mutations in the *BRAF* and *KIT* genes in subsets of melanoma has expanded treatment options to include specific molecularly targeted kinase inhibitors such as vemurafinib and imatinib. The V600E mutation in BRAF has now been associated with approximately 80% of melanomas and seen predominantly in melanomas arising on skin without chronic sun damage [13,14]. Inhibition of mutated BRAF with targeted inhibitor therapy such as vemurafinib has proven successful in achieving complete or partial regression of tumors in many patients, although resistance frequently develops [15]. Our patient demonstrated a 1799T>A (V600E) mutation in his tumor, providing secondary evidence that his tumor most likely arose from a cutaneous lesion. No activating mutations were detected in the kinase, juxtamembrane or extracellular domains the *KIT* gene, consistent with the observation that *BRAF* and *KIT* mutation are associated with different subsets of melanoma and are generally mutually exclusive [13].

## Conclusions

In summary, we describe the first report of metastatic melanoma of the esophagus arising in a background of BE with molecular analysis for the presence of *BRAF* and *KIT* mutations. Our report emphasizes the rarity of the collision between a separate malignancy and BE and demonstrates the utility of molecular studies both for predictive value and, in the case of melanoma, for supporting the conclusion of whether melanoma represents a primary malignancy versus metastases.

## Consent

Written informed consent was obtained from the patient for publication of this case report and any accompanying images. A copy of the written consent is available for review by the Editor of this journal.

## Competing interests

All authors declare that they have no competing interest.

## Authors’ contributions

DGT provided the diagnostic pathology interpretation for the patient material, conceived of, and wrote the paper. NJS performed the endoscopy, obtained the biopsy material and helped in drafting the paper. SO performed the sequence analysis and interpretation of the *BRAF* and *KIT* mutations and helped in drafting the paper. KW aided in sequence interpretation and helped in drafting the paper. KG provided consultation on the diagnostic pathology and helped in drafting the paper. All authors read and approved the final manuscript.
